# Polymorphisms in *Plasmodium falciparum* Chloroquine Resistance Transporter and Multidrug Resistance 1 Genes: Parasite Risk Factors that Affect Treatment Outcomes for *P. falciparum* Malaria after Artemether-Lumefantrine and Artesunate-Amodiaquine

**DOI:** 10.4269/ajtmh.14-0031

**Published:** 2014-10-01

**Authors:** Meera Venkatesan, Nahla B. Gadalla, Kasia Stepniewska, Prabin Dahal, Christian Nsanzabana, Clarissa Moriera, Ric N. Price, Andreas Mårtensson, Philip J. Rosenthal, Grant Dorsey, Colin J. Sutherland, Philippe Guérin, Timothy M. E. Davis, Didier Ménard, Ishag Adam, George Ademowo, Cesar Arze, Frederick N. Baliraine, Nicole Berens-Riha, Anders Björkman, Steffen Borrmann, Francesco Checchi, Meghna Desai, Mehul Dhorda, Abdoulaye A. Djimdé, Badria B. El-Sayed, Teferi Eshetu, Frederick Eyase, Catherine Falade, Jean-François Faucher, Gabrielle Fröberg, Anastasia Grivoyannis, Sally Hamour, Sandrine Houzé, Jacob Johnson, Erasmus Kamugisha, Simon Kariuki, Jean-René Kiechel, Fred Kironde, Poul-Erik Kofoed, Jacques LeBras, Maja Malmberg, Leah Mwai, Billy Ngasala, Francois Nosten, Samuel L. Nsobya, Alexis Nzila, Mary Oguike, Sabina Dahlström Otienoburu, Bernhards Ogutu, Jean-Bosco Ouédraogo, Patrice Piola, Lars Rombo, Birgit Schramm, A. Fabrice Somé, Julie Thwing, Johan Ursing, Rina P. M. Wong, Ahmed Zeynudin, Issaka Zongo, Christopher V. Plowe, Carol Hopkins Sibley, for the WWARN AL and ASAQ Molecular Marker Study Group

## Abstract

Adequate clinical and parasitologic cure by artemisinin combination therapies relies on the artemisinin component and the partner drug. Polymorphisms in the *Plasmodium falciparum* chloroquine resistance transporter (*pfcrt*) and *P. falciparum* multidrug resistance 1 (*pfmdr1*) genes are associated with decreased sensitivity to amodiaquine and lumefantrine, but effects of these polymorphisms on therapeutic responses to artesunate-amodiaquine (ASAQ) and artemether-lumefantrine (AL) have not been clearly defined. Individual patient data from 31 clinical trials were harmonized and pooled by using standardized methods from the WorldWide Antimalarial Resistance Network. Data for more than 7,000 patients were analyzed to assess relationships between parasite polymorphisms in *pfcrt* and *pfmdr1* and clinically relevant outcomes after treatment with AL or ASAQ. Presence of the *pfmdr1* gene N86 (adjusted hazards ratio = 4.74, 95% confidence interval = 2.29 – 9.78, *P* < 0.001) and increased *pfmdr1* copy number (adjusted hazards ratio = 6.52, 95% confidence interval = 2.36–17.97, *P* < 0.001**)** were significant independent risk factors for recrudescence in patients treated with AL. AL and ASAQ exerted opposing selective effects on single-nucleotide polymorphisms in *pfcrt* and *pfmdr1.* Monitoring selection and responding to emerging signs of drug resistance are critical tools for preserving efficacy of artemisinin combination therapies; determination of the prevalence of at least *pfcrt* K76T and *pfmdr1* N86Y should now be routine.

## Introduction

Recent successes in malaria control have depended on the use of highly efficacious artemisinin combination therapies (ACTs) for first-line treatment of uncomplicated *Plasmodium falciparum* malaria. Adequate clinical and parasitologic cure by ACTs relies on the rapid reduction in parasite biomass by the potent, short-acting artemisinin component^1^–^3^ and the subsequent elimination of residual parasites by the longer-acting partner drug. The two most commonly used ACTs worldwide are artemether-lumefantrine (AL) and artesunate-amodiaquine (ASAQ).^4^ Polymerase chain reaction (PCR)–adjusted efficacy for both combinations remains high in most regions.^5^–^7^ However, there have been some reports of decreasing AL cure rates in Africa^8^–^11^ and Asia,^12^ and reports of high levels of treatment failures of ASAQ.^13^–^18^ Resistance to ACT partner drugs has historically manifested before that of artemisinins, whose short half-lives result in the exposure of residual parasites to sub-therapeutic levels of the partner drug alone. Response to the partner drug is therefore a key component of overall ACT efficacy.

Mutations in the gene encoding the *P. falciparum* chloroquine resistance transporter (*pfcrt*) are associated with chloroquine resistance^19^; a change from lysine to threonine at codon 76 in *pfcrt* predicts responses of parasites to chloroquine.^20^,^21^ In the presence of *pfcrt* 76T, chloroquine resistance is modulated by point mutations in the gene that encodes the *P. falciparum* multidrug resistance transporter 1 (*pfmdr1*), primarily at codon 86^22^,^23^ and also by mutations at positions 184, 1034, 1042, and 1246.^24^ Decreased susceptibility to lumefantrine has been linked to polymorphisms in these two genes.^25^–^35^ Increased *pfmdr1* copy number, which confers resistance to mefloquine,^36^ has also been associated with reduced susceptibility to lumefantrine.^37^–^40^

Some studies of amodiaquine have reported reduced *in vivo* response^41^–^43^ and increased 50% inhibitory concentration values *in vitro*, in association with the presence of *pfmdr1* 86Y and *pfcrt* 76T alleles.^44^,^45^ Selection of these alleles in recurrent parasites after treatment with amodiaquine alone or in combination with artesunate has been observed in a number of studies.^28^,^46^–^51^ It has also been suggested that parasites that carry chloroquine-resistant *pfmdr1* alleles may be more susceptible to artesunate in classical in *vitro* assays,^24^,^52^ an effect that could counteract the increased risk of amodiaquine failure when these drugs are combined in ASAQ.

Currently, AL and ASAQ retain high clinical efficacy with few recrudescent infections, and individual studies generally lack sufficient statistical power to assess the association between parasite genotypes and outcomes of clinical treatment. Such an assessment is a critical step in validating molecular changes in parasite populations as useful markers of early signs of changing parasite susceptibility to lumefantrine or amodiaquine. To overcome these challenges, individual patient data on *in vivo* antimalarial efficacy and molecular markers of *P. falciparum* from 31 clinical trials were standardized, pooled, and > 7,000 patient responses were analyzed to determine whether patients infected with parasites that carry these polymorphisms are at increased risk of treatment failure. This large data set also provided the opportunity to examine the effects of AL and ASAQ treatment on selection in parasites of particular alleles of *pfcrt* and *pfmdr1*.

## Methods

### Selection and inclusion of data.

**Figure 1.**
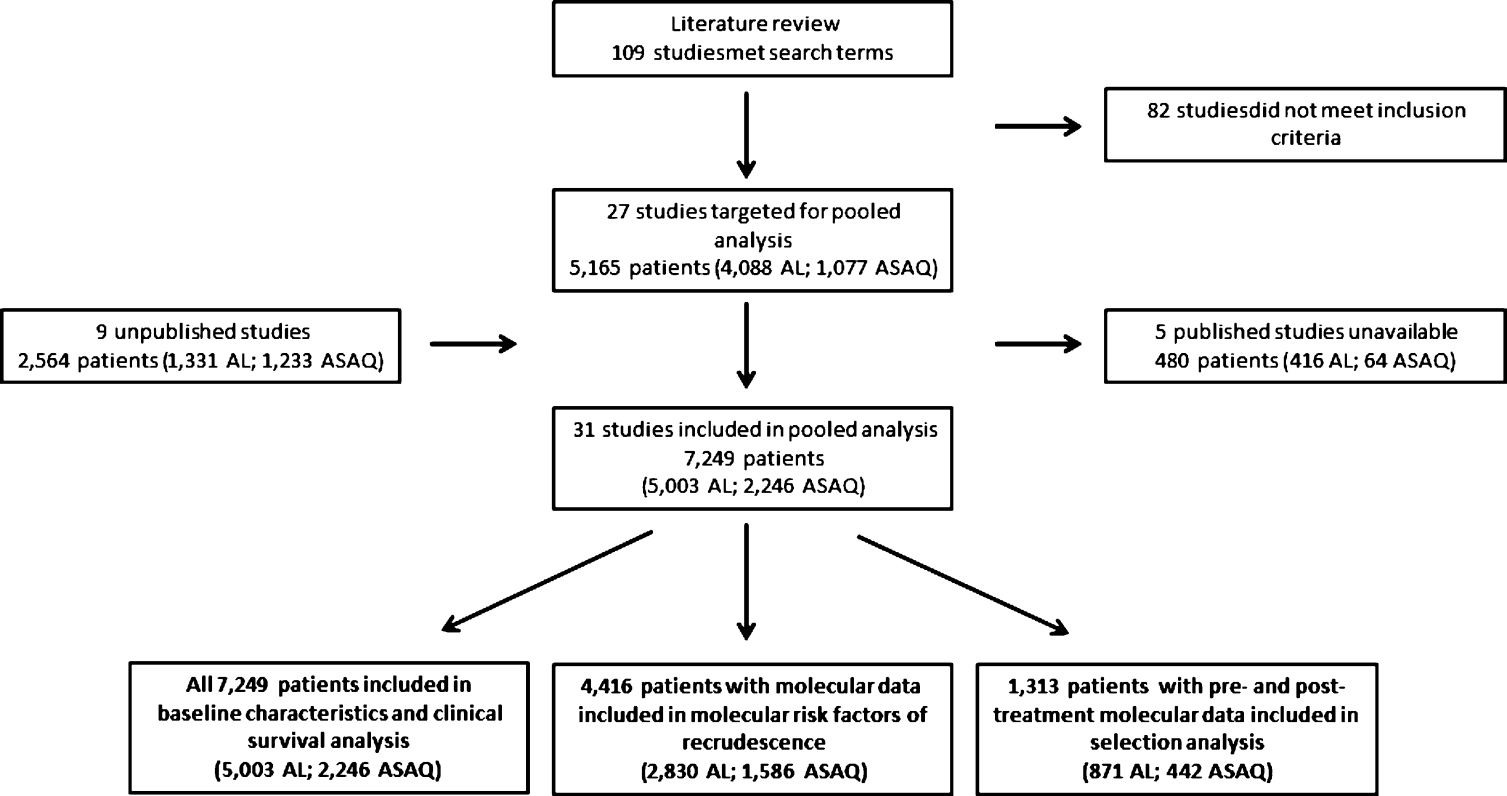
Patient flow chart for study of parasite risk factors that affect treatment outcomes for *Plasmodium falciparum* malaria after treatment with artemether-lumefantrine (AL) or artesunate-amodiaquine (ASAQ).

Prospective clinical efficacy studies of *P. falciparum* treatment with AL (six-dose regimen) or ASAQ (three-day fixed dose or loose/co-blistered regimen) with a minimum of 28 days of follow-up and genotyping of *pfcrt* and/or *pfmdr1* were sought for the analysis. Studies were identified by a systematic PubMed literature review using the search terms (artesunate AND amodiaquine) OR (artemether AND lumefantrine) OR (ACT) AND (*pfmdr1* OR *pfcrt).* Abstracts and text were screened to determine whether inclusion criteria were met. Nine unpublished datasets were also solicited and included in the analysis (see Supplemental Table 3). Individual anonymized patient data including baseline characteristics, drug intake, parasite density and temperature were collected. All but one study included parasite genotyping to identify recrudescent infections of *P. falciparum*, and all studies assessed the presence of *pfcrt* and/or *pfmdr1* polymorphisms (single nucleotide polymorphisms (SNPs) and copy number variation) in parasites isolated from patients on day 0. Multiplicity of infection and molecular resistance marker data from other days including the day of microscopic recurrent parasitemia were included but were not a prerequisite for study inclusion. Metadata on study location, study design, drugs, and dosing regimens were also gathered. A schematic of the patient numbers and overall flow of the study is shown in Figure 1.

### Data curation and generation of variables.

All data sets were uploaded to the WorldWide Antimalarial Resistance Network repository and standardized by using the WorldWide Antimalarial Resistance Network Data Management and Statistical Analysis Plans (DMSAP).^53^,^54^ Outcome status and censoring were defined according to the Clinical DMSAP.^53^ Parasites that recurred within the follow-up period were classified using World Health Organization guidelines^55^: microscopically detected infections during follow-up were classified as recurrent; recurrent infections sharing with blood samples taken at day 0 PCR bands in polymorphic merozoite antigens or microsatellite fragment sizes were classified as recrudescent, and recurrent infections not sharing PCR bands or microsatellite fragment sizes with blood samples taken at day 0 were classified as re-infections (new infections). Molecular markers were coded as either single or mixed allele genotypes in the case of SNPs and as mean copy number per sample for copy number polymorphisms. Multi-SNP haplotypes were reconstructed as described in the Molecular DMSAP.^56^,^57^

### Statistical analysis.

All statistical analyses were conducted by using Stata 11 (StataCorp LP, College Station, TX). The primary endpoint was clinical efficacy, defined as the PCR-adjusted risk of *P. falciparum* recrudescent infections. The cumulative risk of recrudescence at day 28 and day 42 was computed by using survival analysis (Kaplan-Meier estimates [K-M]). Comparisons of K-M survival curves were performed by using log rank tests stratified by study sites.

Multivariable analysis of risk factors associated with PCR-adjusted recrudescence was conducted by using Cox proportional hazards regression models with shared frailty parameters to adjust for site-specific effects. The risk factors that affect the clinical efficacy of AL and ASAQ have been intensively studied in pooled analyses of both ACTs. Sixty-two studies with 14,679 patients treated with AL and 39 studies with 7,652 patients treated with ASAQ were analyzed; these full analyses have been submitted for publication. The univariable and multivariable risk factors identified in those studies are shown in Supplemental Tables 1 and 2. Clinical covariates in the current study were included based on the previous analyses as follows: (lumefantrine or amodiaquine dose, enrolment parasitemia, age category, and ASAQ fixed or co-blistered versus loose formulation (Table 1). Each molecular marker was then added to the model. The proportional hazard assumption was tested based on residuals of Schoenfeld.^58^ In the case of non-proportionality, interactions with a categorized time variable based on clinical follow-up intervals (< day 14, days 14–21, 21–28, and > day 28) were used to account for changing effects over time, and neighboring windows with similar effects of genetic covariates as determined by Wald test were merged. Finally, other covariates (transmission intensity, region of sample origin, dose supervision, and fat intake) were included in the model if they improved model fit based on the likelihood ratio test. Multiplicity of infection was only available for 197 and 141 AL and ASAQ patients, respectively, and was excluded from further analysis. The final model was then used to estimate the adjusted hazard ratio for recrudescence in patients who carried parasites with resistant versus sensitive genotypes on day 0. The assumption of proportional hazards was tested separately for the individual covariates in the final multivariable model, and any violations were reported.

In patients who had recurrent parasitemia on or before day 42, changes in *pfcrt* and *pfmdr1* alleles between pre-treatment and post-treatment matched pairs of samples was compared by using McNemar's test. Changes in genotype, rather than presence of a particular allele, were compared between matched pairs to ensure that differences reflected selection rather than underlying differences in allele frequencies among populations. The effect of markers present at the time of recurrence on median time to PCR-adjusted re-infection (new infection) was investigated by using the Wilcoxon Mann-Whitney U test. Competing risk analysis^59^ was used to estimate cumulative incidence of PCR-adjusted re-infections with specific genotypes, where recrudescent and re-infections with other genotypes were treated as competing events.

The number of molecular markers used to distinguish recrudescence from re-infection varied from one to three or more loci. The effect of the number of loci genotyped on outcome classification was investigated in a regression model of predictors of recrudescence within all recurrences. No effect of this variable was observed on the number of recrudescent infections identified among recurrences in univariable or multivariable analysis, it was not further investigated.

## Results

Individual patient and linked parasite genotype data from 31 studies were available (Supplemental Table 3). Data from 7,249 patients who were treated with AL (5,003) or ASAQ (2,246) were included in the analysis. Twenty seven studies were published, representing 91% of all published clinical data on AL and ASAQ in which *pfcrt* or *pfmdr1* genotypes were determined. Baseline characteristics for patients treated with AL or ASAQ are shown in Supplemental Table 4.

### Clinical efficacy of AL and ASAQ.

The estimates of efficacy (defined as risk of PCR-adjusted recrudescence) of AL and ASAQ are shown in Table 2. Of the 5,003 AL patients, 4,763 were followed-up for at least one day and were included in the analysis. Similarly, of the 2,246 ASAQ patients, 2,099 were included. In total, 1,107 patients had recurrent parasitemia after treatment with AL, of which 188 (18%) were classified by PCR as having recrudescent infections. The corresponding figures for ASAQ showed that 484 patients had recurrent parasitemia and 58 (12%) were confirmed as having recrudescent infections. The overall clinical efficacy at day 42 was 94.8% (95% confidence interval [CI] = 94–95.5%) in patients treated with AL and 95.1% (95% CI = 92.3–96.7%) in patients treated with ASAQ (Table 2). The proportion of adequate clinical and parasitologic response of ASAQ was significantly higher for the fixed dose and co-blistered tablets (97.0%, 95% CI = 94.4–98.4%) compared with the loose formulation (93.0%, [95% CI = 89.2–95.6) (*P* = 0.003).

### Baseline prevalence of genetic markers associated with resistance.

The baseline prevalence of SNPs in *pfcrt* and *pfmdr1* was determined, but not all SNPs were available for all isolates. The most frequently analyzed SNPs were position 76 in *pfcrt* determined for 3,640 patients and position 86 in *pfmdr1* for 3,580 patients, with the complete haplotype of positions 72–76 in *pfcrt*, *pfmdr1* copy number, and SNPs at positions *pfmdr1* 184, 1034, 1042, and 1246 available in a subset of patients (Table 3).

The prevalence of *pfcrt* and *pfmdr1* alleles varied by region (Table 3). The *pfcrt* 76T allele (all in the SVMNT haplotype) was almost fixed at 96.4% (81/84) in isolates from Asia (Thailand) and Oceania (Papua New Guinea). In Africa, the only resistant haplotype observed was the CVIET allele. The 76T allele predominated: 67.6% (1,155/1,708) in East Africa and 73.3% (1,354/1,848) in West Africa (Table 3). Amplification of *pfmdr1* was seen in 50% (88/176) of isolates from Asia examined for this genotype, but only in 2.6% (17/659) of isolates from Africa. *Pfmdr1* 86Y was found in 29.2% (66/226) of isolates from Asia/Oceania; in contrast, the 86Y allele was present in 61.1% (1247/2033) of isolates from East Africa and 48.7% (643/1321) of isolates from West Africa.

The SNPs at positions 184 and 1246 showed similar patterns, with *pfmdr1* Y184 and D1246 predominating in all three regions (Table 3). Almost all isolates examined carried the *pfmdr1* S1034 (760/844) and N1042 (1,053/1,064).

### Parasite genotypes as risk factors for recrudescent infection.

**Figure 2.**
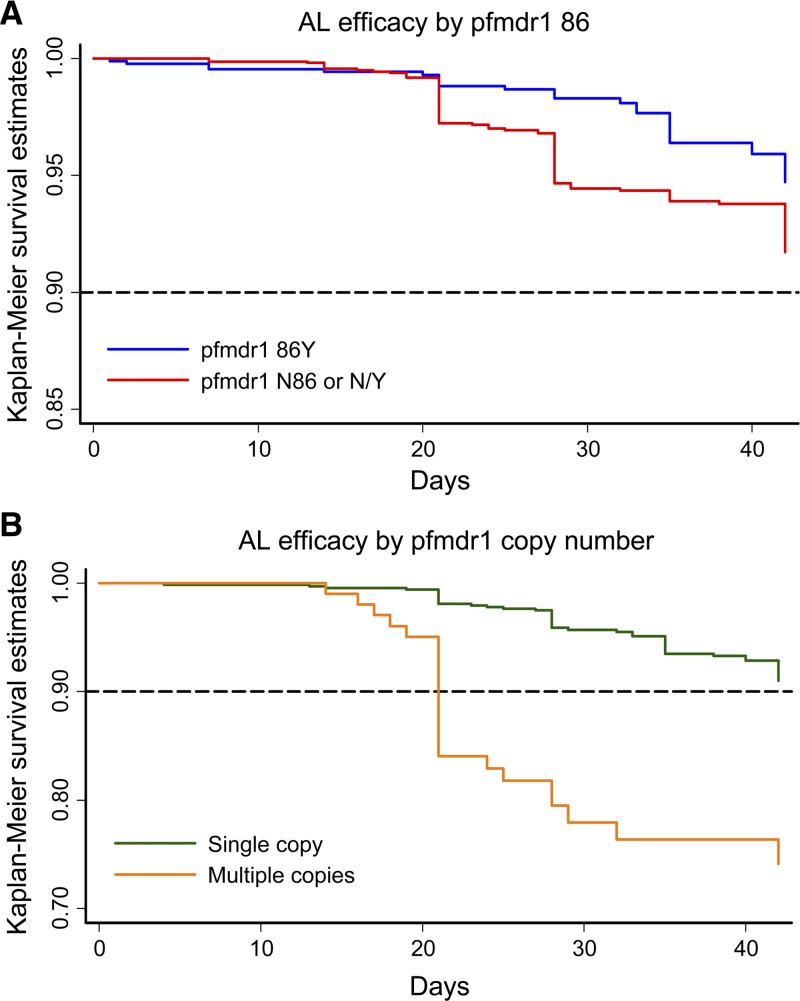
Polymerase chain reaction–adjusted efficacy as assessed by Kaplan-Meier survival estimates for artemether-lumefantrine (AL) by *Plasmodium falciparum* multidrug resistance 1 (*pfmdr1*) genotype of initial parasites. Dotted line indicates World Health Organization–recommended 90% efficacy cutoff value for antimalarial drugs. Clinical response of patients with parasites that carry **A**, *pfmdr1* 86Y (blue) versus 86N or N/Y (red); n = 2,543 patients at risk and **B**, *pfmdr1* copy number > 1 (yellow) versus single copy (green); n = 808 patients.

After controlling for age, baseline parasite density, and total lumefantrine dose (Table 1), the presence of parasites in the initial infection that carried *pfmdr1* N86 (alone or a mixed infection with *pfmdr1* 86Y) was a significant risk factor for recrudescent infection occurring between days 14 and 28 after AL treatment (adjusted hazards ratio [AHR] = 4.74, 95% CI = 2.29–9.78, *P* < 0.001) (Table 4 and Figure 2A). Region of sample origin was not included as a covariate in the model because it violated the assumption of proportional hazards. The risk associated with presence of *pfmdr1* N86 remained significant when excluding infections with multiple copies of *pfmdr1* (AHR = 3.93, 95% CI = 1.90–8.94, *P* < 0.001). The region of sample origin interacted significantly with *pfmdr1* N86, showing that the marker had a larger effect in Asia (AHR = 14.06, 95% CI = 4.52–43.74, *P* < 0.001) than in Africa (AHR = 3.72, 95% CI = 1.77–7.79, *P* = 0.001). However, this interaction violated the proportional hazards assumption since there were so few samples in Africa that had multiple copies of *pfmdr1*, and this variable was excluded from the final model.

The presence of more than one copy of *pfmdr1* was a significant risk factor for recrudescence occurring between days 14 and 21 after AL treatment (AHR = 5.81, 95% CI = 2.38–14.21, *P* < 0.001) (Figure 2B). When the effect of region of origin was added to the model, patients with parasites carrying multiple copy numbers of *pfmdr1* were associated with an increased risk of recrudescence before day 14 (AHR = 83.56, 95% CI = 7.43–939.70, *P* < 0.001) as well as between days 14 and 21 (AHR = 18.54 (95% CI = 7.61–45.19, *P* < 0.001) (Table 4). The interaction of region of origin with *pfmdr1* copy number could not be investigated because of insufficient multicopy samples from Africa in the model.

When *pfmdr1* N86 and *pfmdr1* copy number were included in the same model, region of sample origin was no longer a significantly predictive covariate in the multivariable analysis or as an interaction term with either genotype. Both markers remained as significant predictors of recrudescent infection, between days 14 and 28 for *pfmdr1* N86 (AHR = 5.98, 95% CI = 1.68–21.36, *P* = 0.006) and days 14 and 21 for multiple copies of *pfmdr1* (AHR = 6.52, 95% CI = 2.36–17.97, *P* < 001); Table 4).

No association was observed between the *pfmdr1* 184, *pfmdr1* 1246, and *pfcrt* polymorphisms and recrudescent infections after AL treatment. The risk for parasites with the *pfmdr1* N86 + D1246 haplotype is not reported here because it represents a subset of the *pfmdr1* N86 sample set (of the samples genotyped for both SNPs, all but 17 samples with *pfmdr1* N86 also had D1246). For patients treated with ASAQ, none of the analyzed *pfcrt* or *pfmdr1* parasite genotypes were significant risk factors for recrudescent infections in the multivariable analysis.

### Post-treatment selection of genetic markers associated with resistance.

To examine changes in the genotypes of parasites after drug treatment, we compared the prevalence of *pfmdr1* and *pfcrt* alleles in paired isolates from the initial and the recurrent parasites in the subset of patients in whom parasites recurred during the 42 day follow-up period. Post-treatment changes among specific genotypes are shown in Table 5 for all recurrent infections. Significant selection of *pfcrt* K76, and *pfmdr1* N86 occurred in recrudescent and re-infecting parasites after AL treatment. Selection of *pfmdr1* 184F and D1246 alleles was also observed in the recurrent parasites and *pfmdr1* D1246 in those that reinfected patients after treatment. Selection of single or multiple copies of *pfmdr1* was not observed in any of the groups (Table 5). *Pfmdr1* 86Y and 1246Y were significantly selected in recurrent and re-infections after treatment with ASAQ (Table 5).

### Median time to re-infection.

**Figure 3.**
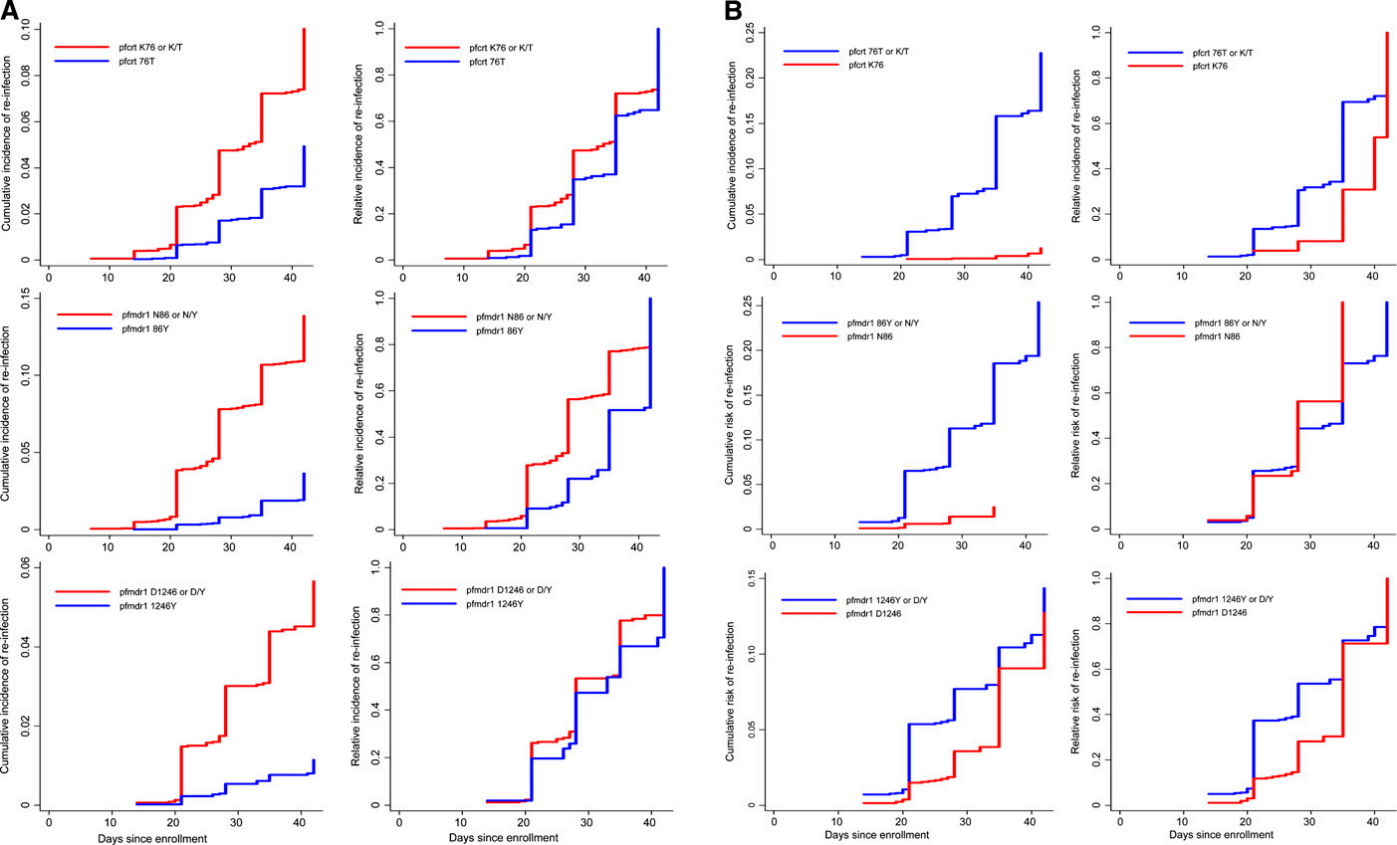
**A**, Cumulative (left panels) and relative (right panels) risks of polymerase chain reaction (PCR)–adjusted reinfection for baseline *Plasmodium falciparum* chloroquine resistance transporter (*pfcrt*) and *P. falciparum* multidrug resistance 1 (*pfmdr1*) genotypes after artemether-lumefantrine treatment, in which recrudescent and re-infections with other genotypes were treated as competing events. **B**, Cumulative (left panels) and relative (right panels) risks of PCR-adjusted re-infection for baseline *pfcrt* and *pfmdr1* genotypes after artesunate-amodiaquine treatment, in which recrudescent and re-infections with other genotypes were treated as competing events.

The genotype of parasites at the time of re-infection provides another metric of their susceptibility to a drug. This analysis indicated that in patients treated with AL, re-infecting parasites carrying *pfmdr1* N86, *pfmdr1* D1246, or *pfcrt* K76 alleles appeared earlier than those carrying *pfmdr1* 86Y, *pfmdr1* 1246Y, or *pfcrt* 76T (Figure 3A). Correspondingly, in patients treated with AL, parasites carrying *pfmdr1* N86 had a median time to re-infection of 28 days (interquartile range = 21–35 days) compared with 35 days (interquartile range = 28–42 days) for those with *pfmdr1* 86Y (*P* < 0.001). Similar differences in the time to re-infection were observed for patients infected with parasites that carried the *pfmdr1* 184F (*P* = 0.008) or *pfcrt* K76 alleles (*P* = 0.001) compared with *pfmdr1* Y184 or *pfcrt* 76T.

In contrast, in patients treated with ASAQ, parasites carrying *pfmdr1* 86Y, *pfmdr1* 1246Y, or *pfcrt* 76T appeared earlier after treatment than those carrying *pfmdr1* N86, *pfmdr1* D1246 or *pfcrt* K76 (Figure 3B). Parasites with *pfcrt* 76T had a median reinfection day of 28 (interquartile range = 21–35) compared with day 37.5 (interquartile range = 28–42) for those carrying K76 (*P* = 0.053) and those with *pfmdr1* 1246Y re-infected on a median day of 21 (interquartile range = 21–28) compared with day 28 (interquartile range = 21–35) for those with D1246 (*P* = 0.001).

## Discussion

This pooled analysis of data from 31 clinical studies shows clearly that the genotypes of infecting parasites influence the outcome of AL treatment. Patients infected with parasites that carried the *pfmdr1* N86 allele or increased *pfmdr1* copy number were at significantly greater risk of treatment failure than those whose parasites carried the 86Y allele or a single copy of *pfmdr1*. Analysis of the clinical outcomes after treatment with ASAQ did not link a particular genotype with treatment failure in this smaller data set. However, it did show clear evidence of selection of particular parasite genotypes. Our findings are consistent with those of previous molecular studies in which changes in the prevalence in the parasite population of particular alleles of *pfcrt* or *pfmdr1* have been documented in response to introduction or increased use of lumefantrine 25–35 or amodiaquine.^15^,^28^,^40^–^51^

Our observation that parasites with the *pfmdr1* N86, D1246, and *pfcrt* K76 alleles re-infected patients earlier after AL treatment, and parasites carrying the *pfmdr1* 86Y, 1246Y, and *pfcrt* 76T alleles re-infected patients earlier after ASAQ is also congruent with the molecular studies. These differences suggest that parasites with these genotypes can withstand higher drug concentrations compared with parasites that carry the alternative alleles. Recently, Malmberg and others^33^ demonstrated this effect quantitatively. After AL treatment, parasites with the *pfmdr1* N86/184F/D1246 haplotype were able to re-infect patients whose lumefantrine blood concentrations were 15-fold higher than was the case for parasites carrying the 86Y/Y184/1246Y haplotype,^33^ providing a potential pharmacologic explanation for the molecular findings. Together, these observations suggest that monitoring shifts to earlier time of re-infection could provide a relatively simple warning of decreasing susceptibility to these drugs, especially if combined with timed measurement of drug concentrations in patients' blood.

In Southeast Asia, parasites with increased *pfmdr1* copy number are common in areas where mefloquine has been intensively deployed,^36^ and almost half of the samples in our data set from that region had at least two copies of the gene. Increased *pfmdr1* copy number was rarely observed in our large sample of isolates from Africa, populations that have had little exposure to mefloquine. Lumefantrine has a shorter half-life in patients than mefloquine,^60^ and may not exert an equivalently strong selection for copy number increase. However, in areas where mefloquine is being introduced, close attention to *pfmdr1* copy number is clearly warranted. A recent report of parasites in Ghana with increased *pfmdr1* copy number underscores the importance of including this parameter in molecular surveillance.^61^

This study supported the conclusion that parasites with increased copy number of *pfmdr1* are also less sensitive to lumefantrine.^37^–^40^ In Southeast Asia, the amplified alleles almost always carried the N86 allele of *pfmdr1*.^34^,^36^,^62^ However, this was not the case in the few parasites from Africa in our data set that did have an increased copy number^31^ so either of the N86Y alleles of *pfmdr1* can apparently be amplified. It is also important to note that increased copy number and the presence of the *pfmdr1* N86 allele were independent risk factors for treatment failure in our analysis.

The evidence of strong selection of particular alleles by both drugs in recurrent parasites, coupled with our observation that particular parasite genotypes increase risk of treatment failure, demonstrates that tracking these molecular markers can signal early decreases in susceptibility to lumefantrine or amodiaquine. Both alleles of *pfmdr1* N86Y, Y184F, and D1246Y are common in *P. falciparum* populations in Africa, and *pfcrt* K76 has increased in prevalence in recent years. Thus, changes in the prevalence of these alleles can be a sensitive indicator of selection of parasite populations by AL and ASAQ. In turn, decreasing efficacy of these partner drugs exposes the artemether or artesunate component of the ACT to selective pressure and could facilitate emergence of new foci of resistance to artemisinin, as observed in the Mekong region. The recent identification of a marker correlated with slow response to artemisinin,^63^ will also enable molecular assessment of this trend.

Application of these molecular tools is increasingly feasible in the context of clinical trials and in community surveys of populations where AL or ASAQ are heavily used. These approaches can offer cost-effective methods that detect evidence of declines in parasite susceptibility far earlier than before, enabling detailed studies of clinical responses to the drugs in areas of concern. This two-stage approach can provide an opportunity for policy makers to manage emerging threats of resistance before clinical failure of a drug is manifest and preserve the useful therapeutic life of these valuable antimalarial drugs for as long as possible.

Finally, these results suggest that AL and ASAQ interact with the proteins encoded by *pfcrt* and *pfmdr1*, but the two drugs select alternative alleles. Two recent publications have also demonstrated that piperaquine exerts selection pressure on these genes in the same direction as amodiaquine, suggesting that the newer ACT, dihydroartemisinin-piperaquine could also function as a counterweight to lumefantrine.^64^,^65^ This opposing selection of parasite genotypes by the partner drugs could influence the choice of an ACT in regions with different patterns of *pfcrt* and *pfmdr1* polymorphisms. For example, if a particular allele is rapidly increasing under intensive use of AL, ASAQ or dihydroartemisinin-, piperaquine might be introduced to counteract that trend. Concurrent use of two ACTs that exert opposing selective pressures on recurrent parasites could provide a counterbalance and prevent strong directional selection in *pfcrt* and *pfmdr1*, maintaining the overall efficacy of AL and ASAQ for a long period. Despite logistical challenges, the simultaneous use of multiple first line therapies is supported by mathematical models,^66^–^68^ and concurrent availability of AL and ASAQ, as implemented in some countries in West Africa^4^ may provide a practical means to test this strategy directly.

## Supplementary Material

Supplemental Tables.

## Figures and Tables

**Table 1:** Multivariable risk factors for PCR-adjusted recrudescent infections for persons treated with artemether-lumefantrine and artesunate-amodiaquine at day 42*

Treatment and variable	Adjusted HR [95% CI]	*P*
AL (n = 14,679; 371 recrudescences)		
Age category: ≥ 12 years (reference)		
< 1	1.55 (0.86–2.78)	0.150
1 to < 5	**2.38 (1.51–3.75)**	**< 0.001**
5 to < 12	1.39 (0.86–2.23)	0.160
Enrollment parasite density (log scale)	**1.13 (1.05–1.23)**	**0.002**
Lumefantrine dose (mg/kg)	1.00 (0.99–1.01)	0.860
ASAQ (n = 7,652; 220 recrudescences)		
Age category: ≥ 12 years (reference)		
< 1	**2.20 (1.01–4.78)**	**0.047**
1 to < 5	**2.27 (1.13–4.55)**	**0.021**
5 to < 12	1.51 (0.72–3.17)	0.140
Enrollment parasite density (log scale)	**1.50 (1.16–1.93)**	**0.002**
Amodiaquine dose (mg/kg)	0.92 (0.82–1.04)	0.180
Drug formulation: fixed dose (reference)		
Co-blistered	0.98 (0.41–2.32)	0.960
Loose	**2.94 (1.58–5.48)**	**0.001**

* Risk factors were selected based upon previous analysis of the same data set (“The effect of dosing strategies on the antimalarial efficacy of artemether-lumefantrine: a pooled analysis of individual patient data, by the WWARN AL Study Group” presubmission approved at PLoS Medicine, March 28, 2014 and “The Effect of Dosing Strategies on the Therapeutic Efficacy of Artesunate Amodiaquine for uncomplicated malaria: A Pooled Analysis of Individual Patient Data” in preparation). Values in bold are statistically significant. PCR = polymerase chain reaction; HR = hazards ratio; CI = confidence interval; AL = artemether-lumefantrine; ASAQ = artesunate-amodiaquine.

**Table 2:** PCR-adjusted adequate clinical and parasitologic response for patients treated with of artemether-lumefantrine and artesunate-amodiaquine after 42 days of follow-up*

Variable	AL	ASAQ fixed dose and co-blistered	ASAQ loose
No. at risk	4,763	1,113	986
ACPR by group, % (95% CI)			
Age category, years			
< 1	96.7 (92.7–98.5)	100	85.2 (70.5–93.0)
1 to < 5	93.6 (92.0–94.8)	96.4 (93.2–98.1)	93.8 (90.0–96.2)
5–12	96.3 (94.5–97.5)	98.8 (91.6–99.8)	99 (96.1–99.8)
≥ 12	95.2 (93.8–96.3)	–	–
Region			
Asia/Oceania	95.2 (93.8–96.2)	–	–
East Africa	93.8 (92.4–95.0)	100†	91.2 (88.0–94.7)
West Africa	96.2 (94.6–97.3)	96.9 (94.2–98.3)	99.2 (96.8–99.8)†
Overall	94.8 (94.0–95.5)	97.0 (94.4–98.4)	93.0 (89.2–95.6)

* PCR = polymerase chain reaction; ACPR = adequate clinical and parasitologic response; AL = artemether-lumefantrine; ASAQ = artesunate –amodiaquine; CI = confidence interval.

† Followed-up to day 28.

**Table 3:** Baseline (pre-treatment) prevalence of genetic markers associated with drug resistance*

Marker	Asia/Oceania	East Africa	West Africa
*pfcrt* 76			
Sample size	84	1,708	1,848
K	3 (4)	553 (32)	494 (27)
K/T	2 (2)	125 (7)	249 (13)
T	79 (94)	1,030 (60)	1105 (60)
*pfcrt* 72–76			
Sample size	84	155	84
CVMNK	3 (4)	37 (24)	14 (17)
CVIET	0	117 (75)	53 (63)
SVMNT	79 (94)	0	0
Mixed	2 (2)	1 (1)	17 (20)
*pfmdr1* 86			
Sample size	226	2,033	1,321
N	160 (71)	759 (37)	678 (51)
N/Y	0	378 (19)	190 (14)
Y	66 (29)	896 (44)	453 (34)
*pfmdr1* 184			
Sample size	228	1,275	686
Y	183 (80)	803 (63)	287 (42)
Y/F	8 (4)	130 (10)	77 (11)
F	37 (16)	342 (27)	322 (47)
*pfmdr1* 1246			
Sample size	77	1,017	687
D	67 (87)	454 (45)	526 (77)
D/Y	10 (13)	309 (30)	86 (13)
Y	0	254 (25)	75 (11)
*pfmdr1* 86 + 1246			
Sample size	69	1,000	685
N D	12 (17)	129 (13)	263 (38)
N Y	0	9 (1)	2 (0)
Y D	50 (72)	248 (25)	199 (29)
Y Y	0	220 (22)	71 (10)
Mixed	7 (10)	394 (39)	150 (22)
*pfmdr1* copy number			
Sample size	176	659	0
1	88 (50)	642 (98)	0
2	57 (32)	16 (2)	0
> 2	31 (18)	1 (0)	0

* Values are no. (%). *pfcrt* = *Plasmodium falciparum* chloroquine resistance transporter gene; *pfmdr1* = *P. falciparum* multidrug resistance 1 (*pfmdr1*) gene.

**Table 4:** Multivariable risk factors for PCR-adjusted recrudescent infections of persons treated with artemether-lumefantrine on day 42*

Marker and variable	Adjusted hazard ratio (95% CI)	*P*
*pfmdr1* 86 (n = 2,543; 135 recrudescent infections)†		
*pfmdr1* N86 or N/Y		
In recrudescence up to day 14	0.79 (0.25–2.54)	0.694
In recrudescence between days 14 and 28	**4.74 (2.29–9.78)**	**< 0.001**
In recrudescence after day 28	0.84 (0.43–1.66)	0.624
Enrollment parasite density (log_e_ – scale)	1.13 (0.99–1.29)	0.056
Age category (reference < 1 year)		
1 to < 5	1.05 (0.40–2.75)	0.922
5 to < 12	0.85 (0.30–2.38)	0.752
≥ 12	0.77 (0.25–2.36)	0.647
Lumefantrine dose (mg/kg)	0.99 (0.98–1.00)	0.109
*pfmdr1* copy number (n = 808; 73 recrudescent infections)		
*pfmdr1* copy number > 1‡		
In recrudescence up to day 14	**83.56 (7.43–939.70)**	**< 0.001**
In recrudescence between days 14 and 21	**18.54 (7.61–45.19)**	**< 0.001**
In recrudescence after day 21	0.61 (0.25–1.51)	0.286
Region (reference Africa)		
Asia/Oceania	**5.09 (1.06–24.38)**	**0.042**
Enrollment parasite density (log_e_ – scale)	1.00 (0.85–1.18)	0.978
Age category (reference < 5 years)		
5 to < 12	0.62 (0.22–1.77)	0.368
≥ 12	0.56 (0.16–1.93)	0.359
Lumefantrine dose (mg/kg)	0.98 (0.96–1.00)	0.113
*pfmdr1* 86 and copy number (n = 719; 59 recrudescent infections)§		
*pfmdr1* N86 or N/Y		
In recrudescence up to day 14	1.00 (0.07–13.64)	0.997
In recrudescence between days 14 and 28	**5.98 (1.68–21.36)**	**0.006**
In recrudescence after day 28	0.51 (0.18–1.47)	0.21
*pfmdr1* copy number > 1		
In recrudescence up to day 14	2.17 (0.16–29.77)	0.561
In recrudescence between days 14 and 21	**6.52 (2.36–17.97)**	**< 0.001**
In recrudescence after day 21	0.94 (0.31–2.82)	0.916
Enrollment parasite density (log_e_ – scale)	1.08 (0.92–1.28)	0.348
Age category (reference < 5 years)		
5 to < 12	1.46 (0.59–3.57)	0.413
≥ 12	0.79 (0.27–2.33)	0.663
Lumefantrine dose (mg/kg)	0.98 (0.95–1.00)	0.05

* Values in bold are statistically significant. PCR = polymerase chain reaction; CI = confidence interval; *pfmdr1* = *P. falciparum* multidrug resistance 1 (*pfmdr1*) gene.

† Region not included as a covariate or interaction term with *pfmdr1* 86 genotype because proportional hazards assumption was not met.

‡ Sparse data for *pfmdr1* copy number in Africa prevented the inclusion of region as an interaction term.

§ Region as a covariate and region-genotype interaction terms did not have statistically significant effects in this model.

**Table 5:** Selection of *pfcrt* and *pfmdr1* genotypes after treatment with artemether-lumefantrine and artesunate-amodiaquine*

Marker	Genotype	Recurrence		Recrudescence		Re-infection	
		AL	ASAQ	AL	ASAQ	AL	ASAQ
*pfcrt 76*	K → T†	16% (89/571)	10% (25/237)	5% (4/73)	20% (7/35)	17% (82/493)	9% (17/196)
	T → K	**30**% **(171**/**571)**	8% (18/237)	**25**% **(18**/**73)**	11% (4/35)	**31**% **(152**/**493)**	7% (14/196)
	No change	54% (311/571)	82% (194/237)	70% (51/73)	69% (24/35)	53% (259/493)	84% (165/196)
*P* value		**< 0.001**	0.286	**0.004 (exact)**	0.366	**< 0.001**	0.590
*pfmdr1 86*	N → Y	13% (95/712)	**27**% **(92**/**341)**	10% (10/101)	18% (5/28)	14% (85/609)	**28**% **(87**/**308)**
	Y → N	**40**% **(286**/**712)**	16% (54/341)	**31**% **(31**/**101)**	14% (4/28)	**42**% **(255**/**609)**	16% (49/308)
	No change	46% (331/712)	57% (195/341)	59% (60/101)	68% (19/28)	44% (269/609)	56% (172/308)
*P* value		**< 0.001**	**0.002**	**0.001**	0.739	**< 0.001**	**0.001**
*pfmdr1 184*	Y→ F	**24**% **(74**/**311)**	12% (37/303)	20% (14/69)	12% (3/25)	25% (60/242)	12% (34/273)
	F → Y	16% (51/ 311)	17% (50/303)	14% (10/69)	4% (1/25)	17% (41/242)	18% (49/273)
	No change	60% (186/311)	71% (216/303)	65% (45/69)	84% (21/25)	58% (141/242)	70% (190/273)
*P* value		**0.040**	0.163	0.414	0.625	0.059	0.100
*pfmdr1 1246*	D → Y	14% (38/273)	**32**% **(102**/**317)**	11% (5/44)	39% (11/28)	15% (33/227)	**32**% **(90**/**284)**
	Y → D	**32**% **(86**/**273)**	19% (60/317)	30% (13/44)	14% (4/28)	**32**% **(73**/**227)**	20% (56/284)
	No change	54% (149/273)	49% (155/317)	59% (26/44)	46% (13/28)	53% (121/227)	48% (138/284)
*P* value		**< 0.001**	**0.001**	0.059	0.119	**< 0.001**	**0.005**
*pfmdr1* copy number	1 → 2 or more	1% (2/269)	–	4% (2/53)	–	0	–
	2 or more → 1	1% (3/269)	–	2% (1/53)	–	1% (2/216)	–
	No change	98% (264/269)	–	94% (50/53)	–	99% (214/216)	–
*P* value		1.000 (exact)		1.000 (exact)		0.500 (exact)	

* Values in bold indicate statistically significant selection (*P* < 0.05) by using McNemar's paired test. Those marked exact were tested by using the exact distribution for small sample sizes. A small number of recurrent infections (4 for AL and 6 for ASAQ) were not polymerase chain reaction–adjusted and were excluded from the analysis of recrudescent and re-infections. *pfcrt* = *Plasmodium falciparum* chloroquine resistance transporter gene; *pfmdr1* = *P. falciparum* multidrug resistance 1 (*pfmdr1*) gene; AL = artemether-lumefantrine; ASAQ = artesunate –amodiaquine.

† Each category includes all changes from one allele to another. For example, K → T includes K→ T, K→ K/T, and K/T → T changes.
