# Erratum

**DOI:** 10.4269/ajtmh.14-0031err

**Published:** 2019-03

This notice is from the authors regarding *Am J Trop Med Hyg* 91 (4) by Venkatesan and others (https://doi.org/10.4269/ajtmh.14-0031).

It has come to our attention that 1) 69 out of more than 800 patients treated with AL4 were erroneously included in the original analysis of the associated increased *pfmdr1* copy number on the risk of clinical failure after AL treatment; 2) there was an error in the code for running the Cox regression model for increased copy number of *pfmdr1*.

As a result, the presence of more than one copy of *pfmdr1* was no longer a significant risk factor for recrudescence after AL treatment and did not become a significant risk factor when the effect of region was added to the model. Once adjusted by the study site, AL efficacy in patients infected with parasites carrying multiple copies of *pfmdr1* was not significantly different from the efficacy in patients whose parasites had a single copy of *pfmdr1* with multi-copy infections (see table in the corrected paper *P* = 0.47; Figure 2B).

This change affected only the subgroup analysis of the risk of increased copy number of *pfmdr1* on clinical treatment efficacy. The association of the prevalence of parasites carrying the N86 allele of *pfmdr1* and increased risk of clinical failure after AL remains the same.

The following changes have been made in the online version of the paper available from the WWARN website. (http://www.wwarn.org/sites/default/files/attachments/documents/erratum-full-paper-polymorphisms-pfcrt-pfmdr1-ajtmh-november-2019.pdf).

Changes described below are highlighted in the corrected version.

1. The statement that increased copy number of pfmdr1 is significantly associated with increased risk of treatment failure has been deleted.
2. The results of the model that concern the increased copy number in Table 4 have been modified.
3. Figure 2B has been recalculated with the correct sample numbers, and the difference in treatment failure between the two genotypes is no longer significant. The corrected figure is now in the linked corrected version.
4. The change in the total population of patient isolates analyzed required minor adjustments in the number of isolates assessed. These have been recalculated to reflect the smaller number of isolates, but the risks associated with specific SNPS and haplotypes of *pfmdr1* remain significant
5. In the results on p. 836 of the print version, two paragraphs beginning with “*The presence of more than one copy of pfmdr1….*” have been replaced with the following paragraph:

The presence of more than one copy of pfmdr1 was not a significant risk factor for recrudescence after AL treatment and did not become a significant risk factor when the effect of region was added to the model. Once adjusted by study site, AL efficacy in patients with infections with a single copy of *pfmdr1* was not significantly different from efficacy in patients with multi-copy infections (*P* = 0.47; Figure 2B).

1. In the discussion, beginning on p. 839 of the print version with the paragraph that begins “*In Southeast Asia, parasites with increased copy number of pfmdr1 are common….*” and the next paragraph “*This study supported the conclusion….*” have been replaced with the three paragraphs below.

In Southeast Asia, parasites with multi-copy *pfmdr1* are common in areas where mefloquine has been intensively deployed,^36^ and increased *pfmdr1* copy number is strongly associated with artesunate–mefloquine treatment failures. Almost half of the samples in our data set from Southeast Asia region had at least two copies of the gene. By contrast, multiple copy number was rarely observed in our large sample of isolates from Africa, where populations have had little exposure to mefloquine.

The results of this study did not indicate that parasites with increased copy number of *pfmdr1* are less sensitive to lumefantrine. Our findings contrast with reports of decreased in vitro lumefantrine susceptibility with increased copy number,^37–40^ but support the conclusions of in vivo studies,^38^ which indicate that multi-copy *pfmdr1* is not a risk factor for AL treatment failure.

In our data set from Southeast Asia, all the amplified alleles carried the N86 allele of *pfmdr1*.^34,36,62^ This association was not found in the few parasites from Africa in our data set that did have an increased copy number,^31^ indicating that either of the N86Y alleles of *pfmdr1* can apparently be amplified in this region.

