# Supplementary material for: Polymorphisms in Plasmodium falciparum Chloroquine Resistance Transporter and Multidrug Resistance 1 Genes: Parasite Risk Factors that Affect Treatment Outcomes for P. falciparum Malaria after Artemether-Lumefantrine and Artesunate-Amodiaquine
Source: Am J Trop Med Hyg. 2014 Oct 1;91(4):833–43. doi: 10.4269/ajtmh.14-0031 (PMC4183414; doi:10.4269/ajtmh.14-0031)
Supplement: Supplementary file 1 [file SD3.pdf]

Supplemental Table 1

Univariable and multivariable risk factors for PCR-adjusted recrudescence at day 42 (n = 14,679 and 371 recrudescences) for patients treated with artemether-lumefantrine*

Variable	No.	Univariate analysis Crude HR (95% CI)	*P*	Multivariate analysis Adjusted HR (95% CI)	*P*	Population attributable risk† Frequency	PAR
Age (years)	14,679	0.96 (0.94–0.98)	< 0.001	–	–	–	–
Weight (kg)	14,769	0.98 (0.97–0.99)	< 0.001	–	–	–	–
Lumefantrine dose (mg/kg)	14,769	1 (0.99–1)	0.550	1 (0.99–1.01)	0.860	28.13%	2.26%
Clinical variables							
Baseline parasitemia (log scale)	14,769	**1.15 (1.07–1.25)**	**< 0.001**	**1.13 (1.05–1.23)**	**0.002**	**9.12%**	**4.15%‡**
Baseline parasitemia > 100,000/µL	14,769	1.55 (1.15–2.09)	0.004	–	–	–	–
Baseline gametocytemia	7,659	1.55 (1.04–2.32)	0.031	–	–	–	–
Age category	14,679						
≥ 12 years (reference)							
< 1		1.74 (1.01–3)	0.045	1.55 (0.86–2.78)	0.150	9.01%	5.68%
1 to < 5		**2.69 (1.73–4.17)**	**< 0.001**	**2.38 (1.51–3.75)**	**< 0.001**	**45.72%**	**41.24%‡**
5 to <12		1.51 (0.95–2.38)	0.079	1.39 (0.86–2.23)	0.160	20.63%	9.21%
Weight category	14,769						
≥ 35 kg (reference)							
5 to < 15		2.47 (1.58–3.88)	< 0.001	–	–	–	–
15 to < 25		1.92 (1.21–3.04)	0.005	–	–	–	–
25 to < 35		1.39 (0.75–2.56)	0.300	–	–	–	–
Supervision	14,396						
Full (reference)				–	–	–	–
Partial		0.92 (0.51–1.67)	0.790	–	–	–	–
Unsupervised		1.66 (0.56–4.93)	0.370	–	–	–	–
Co-administration with fat	7,180			–	–	–	–
With fatty meal (reference)							
Without fatty meal		0.91 (0.32–2.61)	0.860	–	–	–	–

* Values in bold are statistically significant. PCR = polymerase chain reaction; HR = hazards ratio; CI = confidence interval; PAR population attributable risk.
† Overall PAR for model: 52.9% calculated as calculated as $1 - \Pi_{r=1}^{R}(1 - PAR_r)$
‡ Cumulative PAR for hyper-parasitemia and age 1 to < 5 years: 43.7%.

Supplemental Table 2

Univariable and multivariable risk factors for PCR-adjusted recrudescence at day 42 (n = 7,652 and 220 recrudescences for final model) for patients treated with artesunate-amodiaquine*

Variable	No. (no.)†	Univariable analysis Crude HR (95% CI)	*P*	Multivariable analysis Adjusted HR (95% CI)	*P*	Population attributable risk‡ Frequency	PAR
Amodiaquine dose (mg/kg) (5 units)	7,652 (220)	0.90 (0.8–1.01)	**0.081**	0.92 (0.82–1.04)	0.180	–	–
Clinical variables							
Parasitemia (log scale)	8,224 (223)	**1.53 (1.19–1.97)**	**< 0.001**	**1.5 (1.16–1.93)**	**0.002**	10.7%	5.5%
Proportion of baseline parasitemia > 100,000/µL	8,224 (223)	**1.54 (1.03–2.30)**	**0.034**	–	–	–	–
Baseline fever (temperature > 37.5°C)	7,847 (212)	0.87 (0.64–1.20)	0.400	–	–	–	–
Baseline hemoglobin level	5,708 (193)	**0.93 (0.86–1.00)**	**0.054**	–	–	–	–
Baseline anemia (hemoglobin level < 10)	5,708 (193)	**1.37 (1.00–1.89)**	**0.050**	–	–	–	–
Baseline gametocyte level	4,258 (91)	1.41 (0.76–2.59)	0.270	–	–	–	–
Species at enrollment							
Pure *P. falciparum* infection (reference)	8,189 (220)						
Mixed infections	35 (3)	1.28 (0.37–4.41)	0.700	–	–	–	–
Sex							
F (reference)	3,755 (106)						
M	4,308 (102)	0.87 (0.66–1.15)	0.340	–	–	–	–
Age category							
≥ 12 yrs (reference)	1,289 (14)						
< 1	693 (32)	**3.52 (1.65–7.5)**	**0.001**	**2.2 [1.01–4.78]**	**0.047**	8.4%	11.1%
1 to < 5	4,816 (158)	**3.48 (1.78–6.82)**	**< 0.001**	**2.27 [1.13–4.55]**	**0.021**	58.5%	46.9%
5 to < 12	1,426 (19)	1.72 (0.84–3.54)	0.140	1.51 [0.72–3.17]	0.280	–	–
Drug formulation							
FDC (reference)	4,212 (78)						
nFDC co-blistered	900 (11)	1.19 (0.51–2.78)	0.700	0.98 [0.41–2.32]	0.960	–	–
nFDC loose	3,112 (134)	**3.00 (1.64–5.50)**	**< 0.001**	**2.94 [1.58–5.48]**	**0.001**	36.3%	41.9%
Treatment supervision	8,334						
Fully (reference)	6,287 (74)						
Partial	1,937 (149)	2.08 (0.79–5.46)	0.140	–	–	–	–

* Values in bold are statistically significant. PCR = polymerase chain reaction; HR = hazards ratio; CI = confidence interval; FDC = fixed-dose combination.
† No. = number of patients (No.); no. = number of PCR-confirmed treatment failures.
‡ Overall PAR for the model accounted by significant variables: 74.0% calculated as $1 - \Pi_{r=1}^{R}(1 - PAR_r)$. For PAR calculation, parasitemia was categorized at 100,000/µL. Variance of the random effect = 0.914. Anemia was not kept for multivariable analysis because of missing values. The coefficients for other covariates remain unaffected with or without anemia in the model. The assumption of proportional hazard held true for overall final multivariable model globally (P = 0.584) and individually for each of the covariates (P > 0.05).

Supplemental Table 3
Summary of studies included in the analysis*

Region, country	Reference	Study year(s)	Treatment (no.)		Transmission zone (no.)		
			AL	ASAQ	High	Moderate	Low
East Africa							
Ethiopia	67	2006	34				34
Ethiopia	68	2008–2009	348				348
Kenya	Unpublished	2007-2008		54			54
Kenya	69	2005	241		241		
Kenya	15	2007		103		103	
Madagascar	70	2006–2007		17	1	15	1
Sudan	31	2006	91				91
Sudan	16	2003		80			80
Tanzania	71	2007–2008	359		359		
Tanzania	11	2007	244		244		
Tanzania	72	2010	108			108	
Tanzania (Zanzibar)	47	2002–2003		208		208	
Tanzania (Zanzibar)	27	2002–2003	200			200	
Tanzania (Zanzibar)	25	2002–2003	†			†	
Tanzania	73	2004	50		50		
Uganda	74	2004–2007	149	149		298	
Uganda	26	2005	204		204		
Uganda	46	2005		204	204		
Uganda	Unpublished	2007–2008	112		112		
West Africa							
Benin	Unpublished	2007	96	95		191	
Burkina Faso	30	2006	188			188	
Burkina Faso	Unpublished	2004–2006		890	890		
Burkina Faso	75	2005	261			261	
Guinea-Bissau	76	2006–2008	191			191	
Liberia	77	2009	150	149	299		
Mali		2009	337		188	77	72
Mali	49	2002–2004		252	252		
Nigeria	78	2007–2008	47	45	92		
Oceania							
Papua New Guinea	79	2005–2007	176			176	
Asia							
Thailand	34	1995–2002	1,417				1,417
Thailand	29	1995–2002	†				†
Total			**5,003**	**2,246**	**3,136**	**2,016**	**2,097**

* AL = artemether-lumefantrine; ASAQ = artesunate-amodiaquine.
†Samples overlap with previous study.

REFERENCES

1. Eshetu T, Berens-Riha N, Fekadu S, Tadesse Z, Gurkov R, Holscher M, Loscher T, Miranda I, 2010. Different mutation patterns of *Plasmodium falciparum* among patients in Jimma University Hospital, Ethiopia. *Malar J 9:* 226.
2. Eshetu T, Abdo N, Bedru KH, Fekadu S, Wieser A, Pritsch M, Loscher T, Berens-Riha N, 2012. Open-label trial with artemether-lumefantrine against uncomplicated *Plasmodium falciparum* malaria three years after its broad introduction in Jimma Zone, Ethiopia. *Malar J 11:* 240.
3. Borrmann S, Sasi P, Mwai L, Bashraheil M, Abdallah A, Muriithi S, Fruhauf H, Schaub B, Pfeil J, Peshu J, Hanpithakpong W, Rippert A, Juma E, Tsofa B, Mosobo M, Lowe B, Osier F, Fegan G, Lindegardh N, Nzila A, Peshu N, Mackinnon M, Marsh K, 2011. Declining responsiveness of *Plasmodium falciparum* infections to artemisinin-based combination treatments on the Kenyan coast. *PLoS One 6:* e26005.
4. Thwing JI, Odero CO, Odhiambo FO, Otieno KO, Kariuki S, Ord R, Roper C, McMorrow M, Vulule J, Slutsker L, Newman RD, Hamel MJ, Desai M, 2009. In-vivo efficacy of amodiaquine-artesunate in children with uncomplicated *Plasmodium falciparum* malaria in western Kenya. *Trop Med Int Health 14:* 294–300.
5. Andriantsoanirina V, Ratsimbasoa A, Bouchier C, Jahevitra M, Rabearimanana S, Radrianjafy R, Andrianaranjaka V, Randriantsoa T, Rason MA, Tichit M, Rabarijaona LP, Mercereau-Puijalon O, Durand R, Menard D, 2009. *Plasmodium falciparum* drug resistance in Madagascar: facing the spread of unusual pfdhfr and pfmdr-1 haplotypes and the decrease of dihydroartemisinin susceptibility. *Antimicrob Agents Chemother 53:* 4588–4597.
6. Gadalla NB, Adam I, Elzaki SE, Bashir S, Mukhtar I, Oguike M, Gadalla A, Mansour F, Warhurst D, El-Sayed BB, Sutherland CJ, 2011. Increased *pfmdr1* copy number and sequence polymorphisms in *Plasmodium falciparum* isolates from Sudanese malaria patients treated with artemether-lumefantrine. *Antimicrob Agents Chemother 55:* 5408–5411.
7. Hamour S, Melaku Y, Keus K, Wambugu J, Atkin S, Montgomery J, Ford N, Hook C, Checchi F, 2005. Malaria in the Nuba Mountains of Sudan: baseline genotypic resistance and efficacy of the artesunate plus sulfadoxine-pyrimethamine and artesunate plus amodiaquine combinations. *Trans R Soc Trop Med Hyg 99:* 548–554.
8. Ngasala B, Malmberg M, Carlsson A, Ferreira P, Petzold M, Blessborn D, Bergqvist Y, Gil J, Premji Z, Bjorkman A, Martensson A, 2011. Efficacy and effectiveness of artemether-lumefantrine after initial and repeated treatment in children < 5 years of age with acute uncomplicated *Plasmodium falciparum* malaria in rural Tanzania: a randomized trial. *Clin Infect Dis 52:* 873–882.
9. Ngasala BE, Malmberg M, Carlsson AM, Ferreira PE, Petzold MG, Blessborn D, Bergqvist Y, Gil JP, Premji Z, Martensson A, 2011. Effectiveness of artemether-lumefantrine provided by community health workers in under-five children with uncomplicated malaria in rural Tanzania: an open label prospective study. *Malar J 10:* 64.

10. Kamugisha E, Jing S, Minde M, Kataraihya J, Kongola G, Kironde F, Swedberg G, 2012. Efficacy of artemether-lumefantrine in treatment of malaria among under-fives and prevalence of drug resistance markers in Igombe-Mwanza, north-western Tanzania. *Malar J 11:* 58.
11. Holmgren G, Hamrin J, Svard J, Martensson A, Gil JP, Bjorkman A, 2007. Selection of *pfmdr1* mutations after amodiaquine monotherapy and amodiaquine plus artemisinin combination therapy in East Africa. *Infect Genet Evol 7:* 562–569.
12. Sisowath C, Ferreira P, Bustamante L, Dahlstrom S, Martensson A, Bjorkman A, Krishna S, Gil J, 2007. The role of *pfmdr1* in *Plasmodium falciparum* tolerance to artemether-lumefantrine in Africa. *Trop Med Int Health 12:* 736–742.
13. Sisowath C, Stromberg J, Martensson A, Msellem M, Obondo C, Bjorkman A, Gil J, 2005. In vivo selection of *Plasmodium falciparum pfmdr1* 86N coding alleles by artemether-lumefantrine (Coartem). *J Infect Dis 191*: 1014–1017.
14. Sisowath C, Petersen I, Veiga MI, Martensson A, Premji Z, Bjorkman A, Fidock DA, Gil JP, 2009. *In vivo* selection of *Plasmodium falciparum* parasites carrying the chloroquine-susceptible pfcrt K76 allele after treatment with artemether-lumefantrine in Africa. *J Infect Dis 199:* 750–757.
15. Baliraine FN, Rosenthal PJ, 2011. Prolonged selection of *pfmdr1* polymorphisms after treatment of falciparum malaria with artemether-lumefantrine in Uganda. *J Infect Dis 204:* 1120–1124.
16. Dokomajilar C, Nsobya SL, Greenhouse B, Rosenthal PJ, Dorsey G, 2006. Selection of *Plasmodium falciparum pfmdr1* alleles following therapy with artemether-lumefantrine in an area of Uganda where malaria is highly endemic. *Antimicrob Agents Chemother 50:* 1893–1895.
17. Nsobya SL, Dokomajilar C, Joloba M, Dorsey G, Rosenthal PJ, 2007. Resistance-mediating *Plasmodium falciparum pfcrt* and *pfmdr1* alleles after treatment with artesunate-amodiaquine in Uganda. *Antimicrob Agents Chemother 51:* 3023–3025.
18. Dahlstrom S, Aubouy A, Maiga-Ascofare O, Faucher JF, Wakpo A, Ezinmegnon S, Massougbodji A, Houze P, Kendjo E, Deloron P, Le Bras J, Houze S, 2014. *Plasmodium falciparum* polymorphisms associated with *ex vivo* drug susceptibility and clinical effectiveness of artemisinin-based combination therapies in Benin. *Antimicrob Agents Chemother 58:* 1–10.
19. Some AF, Sere YY, Dokomajilar C, Zongo I, Rouamba N, Greenhouse B, Ouedraogo JB, Rosenthal PJ, 2010. Selection of known *Plasmodium falciparum* resistance-mediating polymorphisms by artemether-lumefantrine and amodiaquine-sulfadoxine-pyrimethamine but not dihydroartemisinin-piperaquine in Burkina Faso. *Antimicrob Agents Chemother 54:* 1949–1954.
20. Zongo I, Dorsey G, Rouamba N, Tinto H, Dokomajilar C, Guiguemde RT, Rosenthal PJ, Ouedraogo JB, 2007. Artemether-lumefantrine versus amodiaquine plus sulfadoxine-pyrimethamine for uncomplicated falciparum malaria in Burkina Faso: a randomised non-inferiority trial. *Lancet 369:* 491–498.
21. Ursing J, Kofoed PE, Rodrigues A, Blessborn D, Thoft-Nielsen R, Bjorkman A, Rombo L, 2011. Similar efficacy and tolerability of double-dose chloroquine and artemether-lumefantrine for treatment of *Plasmodium falciparum* infection in Guinea-Bissau: a randomized trial. *J Infect Dis 203:* 109–116.
22. Schramm B, Valeh P, Baudin E, Mazinda C, Smith R, Pinoges L, Dhorda M, Boum Y, Sundaygar T, Zolia Y, Jones J, Comte E, Houze P, Jullien V, Carn G, Kiechel J-R, Ashley E, Guerin P, 2013. Efficacy of artesunate-amodiaquine and artemether-lumefantrine fixed-dose combinations for the treatment of uncomplicated *Plasmodium falciparum* malaria among children aged six to 59 months in Nimba County, Liberia: an open-label randomized non-inferiority trial. *Malar J 12:* 251.
23. Djimde A, Fofana B, Sagara I, Sidibe B, Toure S, Dembele D, Dama S, Ouologuem D, Dicko A, Doumbo O, 2008. Efficacy, safety, and selection of molecular markers of drug resistance by two ACTs in Mali. *Am J Trop Med Hyg 78:* 455–461.
24. Falade CO, Dada-Adegbola HO, Ogunkunle OO, Oguike MC, Nash O, Ademowo OG, 2014. Evaluation of the comparative efficacy and safety of artemether-lumefantrine, artesunate-amodiaquine and artesunate-amodiaquine-chlorpheniramine (artemoclo) for the treatment of acute uncomplicated malaria in Nigerian children. *Med Princ Pract 23:* 204–211.
25. Wong RP, Karunajeewa H, Mueller I, Siba P, Zimmerman PA, Davis TM, 2011. Molecular assessment of *Plasmodium falciparum* resistance to antimalarial drugs in Papua New Guinea using an extended ligase detection reaction fluorescent microsphere assay. *Antimicrob Agents Chemother 55:* 798–805.
26. Price RN, Uhlemann AC, van Vugt M, Brockman A, Hutagalung R, Nair S, Nash D, Singhasivanon P, Anderson TJ, Krishna S, White NJ, Nosten F, 2006. Molecular and pharmacological determinants of the therapeutic response to artemether-lumefantrine in multidrug-resistant *Plasmodium falciparum* malaria. *Clin Infect Dis 42:* 1570–1577.
27. Uhlemann AC, McGready R, Ashley EA, Brockman A, Singhasivanon P, Krishna S, White NJ, Nosten F, Price RN, 2007. Intrahost selection of *Plasmodium falciparum pfmdr1* alleles after antimalarial treatment on the northwestern border of Thailand. *J Infect Dis 195:* 134–141.

Supplemental Table 4

Baseline characteristics of patients treated with artemether-lumefantrine or artesunate-amodiaquine*

Treatment, variable	Asia/Oceania	East Africa	West Africa	Overall
AL				
No. (%)	1,593 (31.9)	2,140 (42.8)	1,270 (25.3)	5,003
Study period	1995–2007	2002–2010	2003–2009	1995–2010
Follow-up (days)				
28	19.5%	32.6%	50.8%	33.0%
42	56.2%	39.3%	49.2%	47.2%
43–63	24.4%	28.0%		19.7%
Median age (IQR, range) (years)	18 (10–30, 0.8–70)	3 (2–5, 0.3–81)	4 (3–7, 0.3–61)	5 (3–14, 0.3–81)
< 1	0.1%	7.7%	3.4%	4.2%
1 to < 5	12.1%	59.9%	47.7%	41.6%
5–11	17.4%	18.6%	38.3%	23.2%
≥ 12	70.5%	13.6%	9.7%	30.7%
Missing	0.0%	0.3%	0.9%	0.4%
Baseline parasites/µL geometric mean (95% CI)	5,371 (4,833–5,969)	14,094 (13,015–15,262)	25,066 (23,411–26,838)	12,086 (11,459–12,748)
Supervision				
Full	64.6%	35.8%	62.2%	51.7%
Partial	11.0%	44.4%	34.1%	31.2%
Unsupervised	0.0%	19.8%	0.0%	8.5%
Not stated/unknown	24.4%	0.0%	3.7%	8.7%
Co-administration				
With food	11.0%	20.7%	26.9%	19.2%
Advised to consume fatty food	0.0%	46.0%	0.0%	19.7%
None	0.0%	9.5%	0.0%	4.1%
Not stated	89.0%	23.7%	73.1%	57.0%
ASAQ				
No. (%)		815 (36.3%)	1,431 (63.7%)	2,246
Study period		2002–2008	2002–2009	2002–2009
Follow-up, days				
28		74.5%	82.9%	79.9%
42		25.5%	17.1%	20.1%
Median age (IQR, range) (years)		3 (2–5, 0.4–60)	3 (2–4, 0.4–38)	3 (2–4, 0.4–60)
< 1		8.3%	7.7%	7.9%
1 to < 5		65.3%	78.6%	73.8%
5–11		19.1%	12.6%	15.0%
≥ 12		7.0%	1.0%	3.2%
Missing		0.2%	0.0%	0.1%
Baseline parasites/µL geometric mean (95% CI)		18,412 (16,480–20,569)	15,661 (14,593–16,806)	16,608 (15,635–17,642)
Formulation				
Fixed dose		3.2%	48.1%	31.8%
Non-fixed dose		94.7%	51.9%	67.4%
Supervision				
Full		63.8%	62.2%	62.8%
Partial			17.1%	10.9%
Not stated/unknown		36.2%	20.8%	26.4%

* AL = artemether-lumefantrine; IQR = interquartile range; CI, confidence interval; ASAQ = artesunate-amodiaquine.
